# Erklärbarkeit der altersadjustierten Übersterblichkeit mit den COVID-19-attribuierten Sterbefällen von Januar 2020 bis Juli 2021

**DOI:** 10.1007/s00103-021-03465-z

**Published:** 2021-12-04

**Authors:** Daniel Wollschläger, Irene Schmidtmann, Sebastian Fückel, Maria Blettner, Emilio Gianicolo

**Affiliations:** 1 Institut für Medizinische Biometrie, Epidemiologie und Informatik (IMBEI), Universitätsmedizin der Johannes Gutenberg-Universität Mainz, Langenbeckstr. 1, 55131 Mainz, Deutschland; 2 Statistisches Landesamt Rheinland-Pfalz, Bad Ems, Deutschland; 3 Institute of Clinical Physiology of the Italian National Research Council (IFC-CNR), Lecce, Italien

**Keywords:** Nicht-pharmazeutische Interventionen, Mortalitätsrate, Öffentliche Gesundheit, Surveillance, Evaluation, Non-pharmaceutical interventions, Mortality rate, Public health, Disease surveillance, Evaluation

## Abstract

**Hintergrund:**

Unsicherheiten in der Todesursachencodierung erschweren die Bestimmung der durch COVID-19 verursachten Mortalität. Dagegen ist die altersadjustierte Übersterblichkeit ein robuster Indikator für Auswirkungen der COVID-19-Pandemie auf die öffentliche Gesundheit. Die Übersterblichkeit spiegelt neben COVID-19-Sterbefällen aber potenziell auch negative Folgen der Maßnahmen zur Pandemieeindämmung wider.

**Ziele:**

Diese Studie prüft, ob es in Deutschland von 01/2020 bis 07/2021 eine Übersterblichkeit gab, die nicht durch COVID-19 erklärbar ist, sondern für indirekte Effekte gesundheitspolitischer Maßnahmen auf die Mortalität spricht.

**Methoden:**

Übersterblichkeitstrends im Zeitraum von 01/2020 bis 07/2021, jeweils in den Bundesländern sowie in den Kreisen von Rheinland-Pfalz, wurden auf Konsistenz mit COVID-19 zugeschriebenen Sterbefällen geprüft. Die erwarteten monatlichen Sterbefälle wurden auf Basis der Daten von 2015 bis 2019 vorhergesagt. Dabei wurden die Bevölkerungsstruktur, Lufttemperatur, saisonale Influenzaaktivität sowie zyklische und langfristige Zeittrends berücksichtigt.

**Ergebnisse:**

In 232/304 (76,3 %) Monat-Bundesland- bzw. in 607/684 (88,7 %) Monat-Kreis-Kombinationen lag die COVID-19 zugeschriebene Mortalität innerhalb der 95 %-Vorhersageintervalle für die Übersterblichkeit. Die Rangkorrelation zwischen Übersterblichkeit und COVID-19-attribuierter Mortalität betrug für die Bundesländer 0,42 (95 %-Konfidenzintervall [0,31; 0,53]) und für die Kreise 0,21 (95 %-Konfidenzintervall [0,13; 0,29]).

**Diskussion:**

Die gute Übereinstimmung der räumlich-zeitlichen Übersterblichkeitsmuster mit den COVID-19 zugeschriebenen Sterbefällen ist konsistent mit der Annahme, dass die Maßnahmen zur Eindämmung der COVID-19-Pandemie zwischen 01/2020 und 07/2021 nicht wesentlich zur Übersterblichkeit in Deutschland beigetragen haben.

**Zusatzmaterial online:**

Zusätzliche Informationen sind in der Online-Version dieses Artikels (10.1007/s00103-021-03465-z) enthalten.

## Abstract (en)

**Background:**

Estimating COVID-19 mortality is impeded by uncertainties in cause of death coding. In contrast, age-adjusted excess all-cause mortality is a robust indicator of how the COVID-19 pandemic impacts public health. However, in addition to COVID-19 deaths, excess mortality potentially also reflects indirect negative effects of public health measures aiming to contain the pandemic.

**Objectives:**

The study examines whether excess mortality in Germany between January 2020 and July 2021 is consistent with fatalities attributed to COVID-19 or may be partially due to indirect effects of public health measures.

**Methods:**

Excess mortality trends for the period from January 2020 to July 2021 were checked for consistency with deaths attributed to COVID-19 in both the German federal states and districts of Rhineland-Palatinate. The expected monthly mortality rates were predicted based on data from 2015–2019, taking into account the population demographics, air temperature, seasonal influenza activity, and cyclic and long-term time trends

**Results:**

COVID-19-attributed mortality was included in the 95% prediction uncertainty intervals for excess mortality in 232 of 304 (76.3%) month–state combinations and in 607 of 684 (88.7%) month–district combinations. The Spearman rank correlation between excess mortality and COVID-19-attributed mortality across federal states was 0.42 (95% confidence interval [0.31; 0.53]) and 0.21 (95% confidence interval [0.13; 0.29]) across districts.

**Conclusions:**

The good agreement of spatiotemporal excess mortality patterns with COVID-19 attributed mortality is consistent with the assumption that indirect adverse effects from public health interventions to contain the COVID-19 pandemic did not substantially contribute to excess mortality in Germany between January 2020 and July 2021.

## Hintergrund

Die *Coronavirus-Disease-2019*-(COVID-19-)bedingte Mortalität gehört zu den schwersten Auswirkungen der Pandemie auf die öffentliche Gesundheit und zählt zu den Indikatoren, die Maßnahmen der Gesundheitspolitik leiten. Die Schätzung der direkt durch COVID-19 verursachten Sterblichkeit ist jedoch anfällig für Verzerrungen: Die zur Verfügung stehenden Testkapazitäten für Infektionen mit dem neuartigen Coronavirus (*Severe Acute Respiratory Syndrome Coronavirus 2*, SARS-CoV-2) wie auch die Indikationen für die Testanwendung variierten erheblich über Zeiträume und Regionen, sodass ein unbekannter Anteil der durch COVID-19 verursachten Sterblichkeit unentdeckt geblieben sein könnte [1]. Andererseits können bei Menschen mit SARS-CoV-2-Infektion mehrere Faktoren zusammenwirken und gemeinsam zum Tod beitragen, was die Identifizierung der Haupttodesursache insbesondere bei Personen mit Komorbiditäten erschwert [2]. Verschiedene Länder haben zudem unterschiedliche Kriterien für die Erfassung von COVID-19-Sterbefällen bei bestätigter oder vermuteter SARS-CoV-2-Infektion festgelegt [1], was einen Vergleich der COVID-19-assoziierten Mortalität über geografische Regionen und Zeiträume hinweg behindert [1, 3].

Im Gegensatz dazu ist die Erfassung der Gesamtmortalität nicht beeinträchtigt durch eine potenziell unsichere Zuordnung der Todesursache. Da die Gesamtmortalität objektiv zu ermitteln ist, kann sie für valide Vergleiche zwischen Zeiträumen und geografischen Regionen verwendet werden. Insbesondere lässt sich ermitteln, ob die beobachteten Sterbefälle von den natürlicherweise zu erwartenden im Sinne einer *Übersterblichkeit* abweichen, welche dann der COVID-19-Pandemie zugeschrieben werden kann. Dabei muss jedoch die Bevölkerungsstruktur berücksichtigt werden [4–7], weil der Anteil älterer Altersgruppen mit höherer Hintergrundmortalität regional unterschiedlich ist und aufgrund der sich ändernden Lebenserwartung zeitlichen Trends unterliegt [8]. Auch andere variable Faktoren wie die Lufttemperatur können die Sterblichkeit wesentlich beeinflussen und sollten daher in Vorhersagemodelle einfließen, um eine präzisere Schätzung der erwarteten Mortalität und damit der Übersterblichkeit zu ermöglichen [9, 10].

Die Gesamtmortalität schließt COVID-19-Sterbefälle ein, kann aber gleichzeitig auch indirekte Pandemiefolgen widerspiegeln [1, 11]. Zum Beispiel führten Veränderungen im gesundheitsbezogenen Verhalten und Einschränkungen der Gesundheitsversorgung unter Umständen zu zusätzlichen Sterbefällen [12, 13]. Progredient verlaufende Krankheiten wurden möglicherweise erst in späteren Stadien diagnostiziert, weil Patientinnen und Patienten bei frühen Symptomen keine ärztliche Hilfe in Anspruch nahmen [14, 15]. Darüber hinaus könnte die reduzierte ärztliche Kapazität die Versorgung von Patientinnen und Patienten mit chronischen Erkrankungen wie Diabetes mellitus und Demenz beeinträchtigt haben, die regelmäßige Kontrolluntersuchungen erfordern.

Die Analyse der Diskrepanz zwischen beobachteter und erwarteter Sterblichkeit im Sinne von Übersterblichkeit [1, 4–7, 16] kann allein nicht zwischen direkten und indirekten Auswirkungen der COVID-19-Pandemie unterscheiden. Wir nutzen hier die geografische Heterogenität und die zeitlichen Trends der COVID-19 zugeschriebenen Mortalität, um deren Muster auf Konsistenz mit der Übersterblichkeit zu prüfen. Nicht durch COVID-19 gedeckte Übersterblichkeit wäre ein Indikator für indirekte negative Effekte der gesundheitspolitischen Maßnahmen zur Pandemieeindämmung.

## Material und Methoden

Die *Mortalitätsraten* wurden in Regressionsmodellen mit Daten von 01/2015 bis 07/2021 zur Gesamtmortalität, zur Bevölkerungsgröße, zur Lufttemperatur und zur Aktivität der saisonalen Influenza sowie mit Informationen zur regionalen sozioökonomischen Deprivation modelliert. Die Regressionsmodelle wurden für 2 verschiedene räumliche Auflösungen angepasst: Für die erste Analyse wurden Daten auf Ebene der Bundesländer verwendet. Für die zweite Analyse wurden auf Kreisebene aufgelöste Daten aus Rheinland-Pfalz gewählt.

### Datenquellen

Jährliche Bevölkerungsdaten und monatliche Sterbefälle für die 16 Bundesländer wurden vom Statistischen Bundesamt stratifiziert nach Geschlecht und Altersgruppe (0–64, 65–74, 75–85, 85+ Jahre) zur Verfügung gestellt [17, 18]. Für 2021 wurde die Bevölkerungsvorausberechnung im Szenario *G2-L2-W1* (moderater Anstieg von Geburtenrate und Lebenserwartung, niedriges Wanderungssaldo) verwendet [19]. Die monatlichen Bevölkerungszahlen wurden durch stratumspezifische lineare Interpolation zwischen den benachbarten jährlichen Zahlen berechnet. Die Sterbefälle für 2021 waren vorläufig.

Das Statistische Landesamt Rheinland-Pfalz lieferte Bevölkerungs- und Sterbefallzahlen für die 36 Landkreise bzw. kreisfreien Städte des Landes, stratifiziert nach Geschlecht und Altersgruppe (10 Jahre). Während die Bevölkerungszahlen für 2019 bis 2021 bereits monatlich aufgelöst waren, wurden die monatlichen Zahlen für 2015 bis 2018 durch stratumspezifische lineare Interpolation zwischen benachbarten jährlichen Zahlen berechnet.

Monatliche Daten zu COVID-19-attribuierten Sterbefällen wurden vom Robert Koch-Institut (RKI) auf Basis von Meldungen gemäß Infektionsschutzgesetz (IfSG) zur Verfügung gestellt [20, 21]. Die Sterbefälle wurden dem Ort der meldenden Einrichtung zugeordnet. Bei Daten auf Ebene der Bundesländer war das angegebene Datum das Sterbedatum. Bei Daten auf Kreisebene war das verwendete Datum – wenn verfügbar – das Sterbedatum, sonst das Meldedatum. Das RKI lieferte außerdem monatliche Daten zur saisonalen Influenzaaktivität in jedem Bundesland und Landkreis [22].

Der Deutsche Wetterdienst stellte monatliche Durchschnittstemperaturen für 01/2015 bis 07/2021 getrennt nach Bundesländern zur Verfügung [23]. Die monatlichen Durchschnittstemperaturen in Rheinland-Pfalz wurden für jeden Kreis auf Basis von Daten der Agrarmeteorologie des Dienstleistungszentrums Ländlicher Raum Rheinland-Pfalz berechnet.

Der *German Index of Socioeconomic Deprivation* (GISD) auf Basis von Indikatordaten aus den Jahren 2015 bis 2017 [24] wurde als Maß für die regionale sozioökonomische Deprivation verwendet, die Einkommen, Beruf und Bildung widerspiegelt.

### Statistik

Für die Bundesländer und rheinland-pfälzischen Kreise wurde je ein *Negativ-Binomial-Regressionsmodell* für die Mortalitätsrate angepasst. Grundlage war jeweils ein Datensatz mit den Werten für Sterbefälle und Kovariaten für alle Beobachtungen (*Poisson-Zellen*), die durch die vollständige Kreuzklassifikation von Monat, Region (Bundesland bzw. Kreis), Geschlecht und Altersgruppe definiert waren. Die Regressionsmodelle wurden an die Daten aus den Jahren 2015 bis 2019 angepasst und dann verwendet, um monatliche Vorhersagen als erwartete Mortalität für 01/2020 bis 07/2021 abzuleiten. Die monatliche Übersterblichkeit für 01/2020 bis 07/2021 wurde je Poisson-Zelle als Differenz zwischen den beobachteten und den vorhergesagten Sterbefällen berechnet.

Zu den Kovariaten gehörten das numerische Kalenderdatum, um langfristige Trends zu berücksichtigen, sowie ein Sinus-Cosinus-Paar für den numerischen Kalendermonat, um die saisonale Variabilität mit einer Periode von einem Jahr zu berücksichtigen ([25]; siehe Onlinematerial). Die mittlere Lufttemperatur wurde als *stückweise linearer Spline* mit einem inneren Knoten bei 18 °C einbezogen, analog zur für London identifizierten Dosis-Wirkungs-Beziehung [26, 27]. Das Modell umfasste außerdem das Geschlecht, die Altersgruppe, die saisonale Influenzaaktivität und den Deprivationsindex GISD. Als *Offset* diente der Logarithmus der Personenzeit, je Poisson-Zelle berechnet als Bevölkerung multipliziert mit der Anzahl der Tage des jeweiligen Monats.

Die Regressionsmodelle wurden mithilfe des Bayes-Modellierungspakets brms [28, 29] für R [30] mit 20.000 Monte-Carlo-Ziehungen pro Modell angepasst. Die Punktschätzungen und 95 %-Glaubwürdigkeitsintervalle für Parameterschätzungen wurden aus den 50 %-, 2,5 %- bzw. 97,5 %-Quantilen der Monte-Carlo-Verteilung berechnet. Die Punktschätzungen und 95 %-Vorhersageintervalle (Prädiktionsintervalle, PI) für die erwartete Anzahl der Sterbefälle wurden aus den 50 %-, 2,5 %- bzw. 97,5 %-Quantilen der *Posterior-Verteilung* berechnet, nachdem die Vorhersagen innerhalb jeder Ziehung aggregiert wurden.

Die Assoziation der monatlichen Übersterblichkeit mit der entsprechenden COVID-19-attribuierten Sterblichkeit wurde als Spearman-Rangkorrelation zusammen mit dem 95 %-*BCa-Bootstrap-Konfidenzintervall* (KI) berechnet.

Um zu prüfen, ob die Regressionsmodellierung die Basissterblichkeit ohne Pandemie schätzen kann, wurden die Modelle in einer Sensitivitätsanalyse auch an die Daten von 2015 bis 2018 angepasst und die beobachtete und vorhergesagte Sterblichkeit im Jahr 2019 verglichen (siehe Onlinematerial).

## Ergebnisse

**Figure 1:**
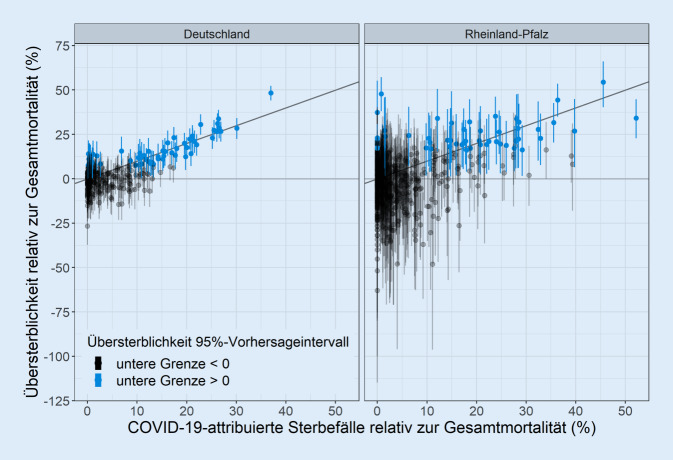

Die geschätzten relativen Raten (RR) spiegelten das saisonale Muster der monatlichen Mortalität sowie einen langsamen Rückgang der Mortalität über die Kalenderjahre wider (siehe Onlinematerial). Erhöhte Sterblichkeit war sowohl mit niedrigen als auch mit hohen Lufttemperaturen assoziiert. Die RR waren für die Altersgruppe 10 bis 19 Jahre am niedrigsten und stiegen für höhere Altersgruppen schnell an. Bei Männern war die Mortalität im Mittel höher als bei Frauen, wobei der Geschlechtsunterschied sowohl in den jungen als auch in den älteren Altersgruppen geringer war. Höhere regionale Deprivation war mit einer höheren Mortalitätsrate assoziiert. Übersterblichkeit und COVID-19-attribuierte Mortalität wiesen einen positiven Zusammenhang auf (Abb. 1).

### Bundesländer

In Deutschland waren im Jahr 2015 29,9 % der Frauen 60 Jahre oder älter. Dieser Anteil stieg auf 31,3 % im Jahr 2020. Der Anteil der Männer im Alter von 60 Jahren oder älter stieg von 24,8 % im Jahr 2015 auf 26,5 % im Jahr 2020.

Insgesamt betrug die Zahl der Sterbefälle in Deutschland von 01/2020 bis 07/2021 1.569.364 mit 90.943 (5,8 %) gemeldeten COVID-19-Sterbefällen. Bezogen auf die Gesamtmortalität lag die Zahl der COVID-19-attribuierten monatlichen Sterbefälle in den verschiedenen Bundesländern zwischen 2,9 % und 10,2 % (Tab. 1). Die entsprechende Übersterblichkeit relativ zur Gesamtsterblichkeit reichte von -3,3 % (95 %-PI [−11,8 %; 4,7 %]) bis 8,9 % (95 %-PI [0,4 %; 16,8 %]).

**Table 1:** 

–		Absolute Todesfälle		Todesfälle/100.000 Bevölkerung		Todesfälle/Gesamtmortalität [%]	
		COVID-19	Übersterblichkeit	COVID-19	Übersterblichkeit	COVID-19	Übersterblichkeit
Deutschland	Total	90.943	48.632 [−73.694; 164.355]	109,3	58,5 [−88,6; 197,5]	5,8	3,1 [−4,7; 10,5]
	Min.	499	−1970 [−15.638; 1473]	55,9	−53,3 [−226,9; 76,9]	2,9	−3,3 [−11,8; 4,7]
	Max.	17.283	10.524 [124; 34.476]	248,9	165,0 [6,7; 360,3]	10,2	8,9 [0,4; 16,8]
Rheinland-Pfalz	Total	3915	832 [−14.557; 15.113]	95,6	20,3 [−355,4; 369,0]	5,0	1,1 [−18,7; 19,4]
	Min.	9	−162 [−662; 182]	26,7	−109,8 [−836,8; 206,7]	1,3	−6,1 [−41,8; 11,8]
	Max.	336	150 [−168; 677]	193,3	260,6 [−227,8; 920,3]	11,2	10,8 [−11,9; 35,1]

**Figure 2:**
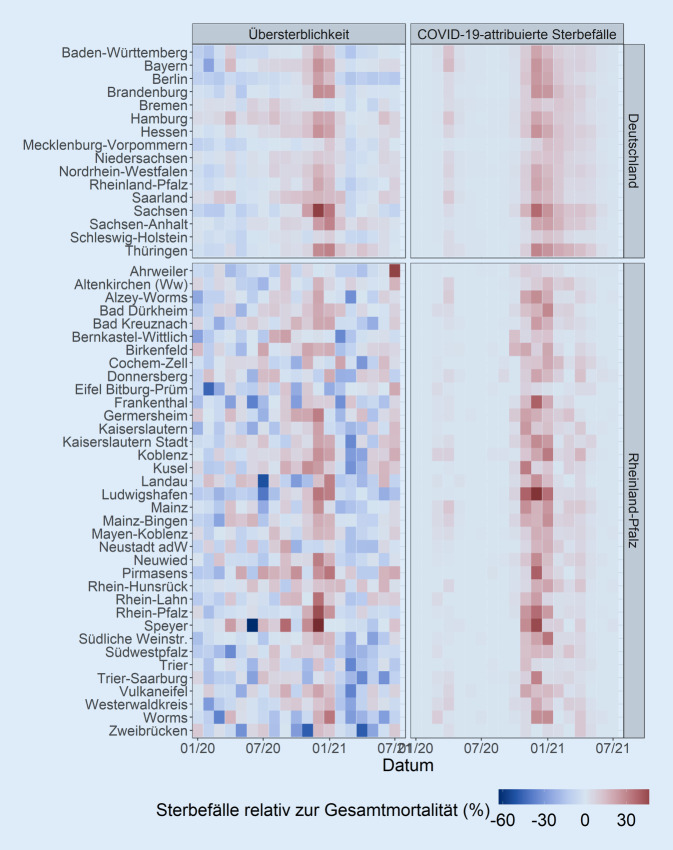

Nur wenige Bundesländer, wie z. B. Bayern, wiesen einen deutlichen Gipfel an COVID-19-attribuierten Sterbefällen während der ersten Welle der Pandemie im März/April 2020 auf (Abb. 2 und 3). Eine größere Anzahl von Bundesländern, wie Brandenburg, Sachsen, Sachsen-Anhalt oder Thüringen, verzeichnete COVID-19-attribuierte Sterbefälle überwiegend während der zweiten Welle im November/Dezember 2020. Im Vergleich zu anderen Bundesländern wurden in Niedersachsen, Schleswig-Holstein und Mecklenburg-Vorpommern sehr wenige COVID-19-attribuierte Sterbefälle während beider Wellen gemeldet.

**Figure 3:**
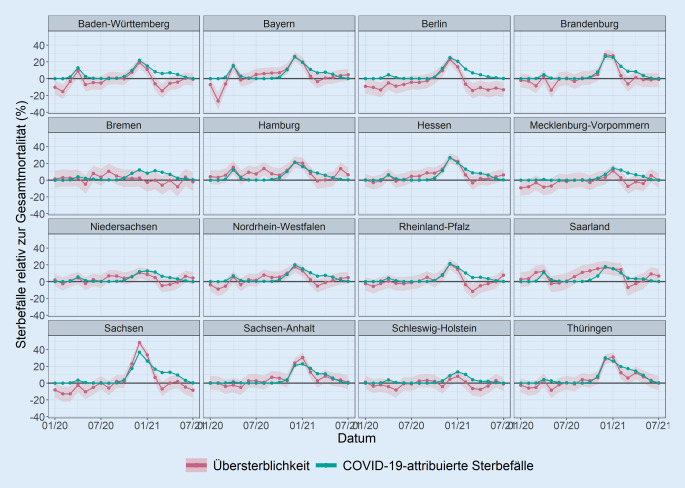

Die Übersterblichkeit folgte generell denselben regionalen und zeitlichen Mustern wie die COVID-19-attribuierten Sterbefälle (Abb. 1, 2 und 3). Die auf COVID-19 attribuierte Mortalität war in 232 von 304 (76,3 %) der Kombinationen aus Kalendermonat und Bundesland in den 95 %-PI für die Übersterblichkeit enthalten. Die Rangkorrelation der monatlichen Übersterblichkeit mit der entsprechenden COVID-19-attribuierten Sterblichkeit betrug 0,42 (95 %-KI [0,31; 0,53]; Abb. 1). Die Rangkorrelation betrug 0,91 (95 %-KI [0,76; 0,96]) beschränkt auf die 57 von 304 Kombinationen von Kalendermonat und Bundesland, bei denen die untere Grenze des 95 %-PI für die Übersterblichkeit über 0 lag. In 228 von 304 (75,0 %) der Beobachtungen war die 0 im 95 %-PI für die Übersterblichkeit enthalten.

Zu den Bundesländern, bei denen die unteren Grenzen der 95 %-PI für die Übersterblichkeit zeitweise höher als die COVID-19-attribuierte Sterblichkeit waren, gehörten Bremen (08/2020), Hamburg (06/2020, 08/2020, 06/2021), Hessen (09/2020), Nordrhein-Westfalen (08/2020), das Saarland (03/2020, 09 bis 11/2020), Sachsen (12/2020, 01/2021) und Sachsen-Anhalt (01/2021). Die Übersterblichkeit war in Hamburg das Jahr 2020 über konstant höher als die COVID-19 zugeschriebene Sterblichkeit. In Bayern überstieg die Übersterblichkeit im Sommer und Herbst 2020 die COVID-19-attribuierte Mortalität, fiel aber noch in die jeweiligen 95 %-PI.

Von 02/2021 bis 07/2021 sank die Übersterblichkeit in vielen Bundesländern deutlich schneller als die COVID-19-attribuierte Mortalität. In allen Bundesländern überschritt die COVID-19 zugeschriebene Mortalität die obere Grenze der 95 %-PI für die Übersterblichkeit 2021 in einigen Monaten, zumeist im März.

### Kreise in Rheinland-Pfalz

In Rheinland-Pfalz waren im Jahr 2015 29,8 % der Frauen 60 Jahre oder älter. Dieser Anteil stieg auf 31,9 % im Jahr 2020. Der Anteil der Männer im Alter von 60 Jahren oder älter stieg von 25,3 % im Jahr 2015 auf 27,6 % im Jahr 2020.

Insgesamt betrug die Zahl der Sterbefälle in Rheinland-Pfalz von 01/2020 bis 07/2021 77.939 bei 3915 (5,0 %) gemeldeten COVID-19-Sterbefällen. Bezogen auf die Gesamtmortalität lag die monatliche Zahl der COVID-19-attribuierten Sterbefälle in den Kreisen von Rheinland-Pfalz zwischen 1,3 % und 11,2 % (Tab. 1). Die entsprechende Übersterblichkeit reichte von −6,1 % (95 %-PI [−41,8 %; 11,8]) bis 10,8 % (95 %-PI [−11,9 %; 35,1 %]) bezogen auf die Gesamtsterblichkeit.

In 607 von 684 (88,7 %) der Kombinationen von Kalendermonat und Kreis war die COVID-19-attribuierte Mortalität in den 95 %-PI für die Übersterblichkeit enthalten. Die Rangkorrelation der monatlichen Übersterblichkeit mit der entsprechenden COVID-19-attribuierten Sterblichkeit betrug 0,21 (95 %-KI [0,13; 0,29]; Abb. 1). Die Rangkorrelation betrug 0,68 (95 %-KI [0,47; 0,80]) beschränkt auf die 56 von 684 Kombinationen von Kalendermonat und Landkreis, bei denen die untere Grenze des 95 %-PI für die Übersterblichkeit über 0 lag. In 583 von 684 (85,2 %) der Beobachtungen war die 0 im 95 %-PI für die Übersterblichkeit enthalten.

Die zeitlichen Trends der Übersterblichkeit auf Kreisebene wiesen größere Schwankungen von Monat zu Monat auf als die der Bundesländer (Abb. 2 und 4). Während viele Kreise während der ersten Welle der Pandemie im März/April 2020 keine erkennbare Übersterblichkeit aufwiesen, zeigten die meisten Kreise während der zweiten Welle im November/Dezember 2020 eine sichtbare Übersterblichkeit und einen gleichzeitigen Anstieg der COVID-19-attribuierten Sterbefälle. Wenige Landkreise, wie Ahrweiler, Eifelkreis Bitburg-Prüm, Trier und Zweibrücken, zeigten auch während der zweiten Welle keinen deutlichen Anstieg der Übersterblichkeit.

**Figure 4:**
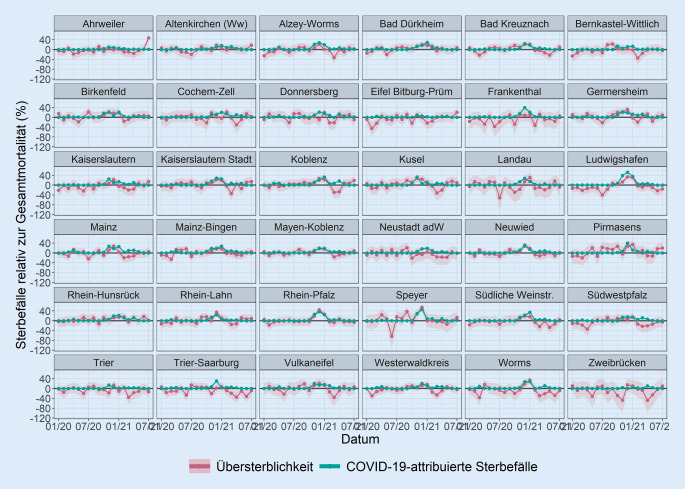

Die Übersterblichkeit folgte generell denselben zeitlichen Trends wie die entsprechenden COVID-19-attribuierten Sterbefälle. Dies war insbesondere der Fall, wenn die COVID-19-attribuierte Sterblichkeit während der zweiten Welle ausgeprägt war, z. B. in Ludwigshafen, im Rhein-Pfalz-Kreis oder in Worms (Abb. 2 und 4). Von Februar bis April 2021 war die Übersterblichkeit in vielen Kreisen negativ und damit niedriger als die COVID-19-attribuierte Mortalität, die sich 0 näherte. In Pirmasens überschritt die untere Grenze der 95 %-PI für die Übersterblichkeit 07/2020, 10/2020 und 01/2021 die COVID-19-attribuierten Sterbefälle. Dies war ebenfalls in Bad Kreuznach (10/2020), Bernkastel-Wittlich (08 bis 09/2020), Birkenfeld (07/2020), Kusel (12/2020), Mainz-Bingen (06/2020), Mayen-Koblenz (08/2020) und Speyer (09/2020) der Fall. Hinzu kamen im Monat der Flutkatastrophe im Ahrtal (07/2021) die Kreise Ahrweiler, Altenkirchen, Koblenz und Bitburg-Prüm.

### Sensitivitätsanalyse

In Deutschland hatte die auf Basis der Daten aus den Jahren 2015 bis 2018 vorhergesagte Zahl der Sterbefälle für 2019 einen mittleren absoluten prozentualen Fehler (MAPE) von 4,3 % über Monate und Bundesländer. Dabei zeigte sich eine Überschätzung der Sterbefälle von 01 bis 05/2019 sowie 12/2019 für die Altersgruppe unter 65 Jahre (siehe Onlinematerial). Die Gesamtdifferenz der vorhergesagten zu den beobachteten Sterbefällen betrug -18.994 und damit 2,0 % der Gesamtsterblichkeit 2019 in Deutschland. Die vorhergesagten Sterbefälle waren 2019 für Hamburg und das Saarland durchweg zu niedrig, wobei in 11 Monaten jeweils die 0 in den 95 %-PI enthalten war. In Berlin waren die vorhergesagten Sterbefälle durchgängig zu hoch, wobei die 0 nur in 2 Monaten in den 95 %-PI enthalten war.

Für Rheinland-Pfalz betrug der mittlere absolute prozentuale Fehler (MAPE) der Vorhersage der Sterbefälle 2019 9,9 % über Monate und Kreise. Die Gesamtdifferenz betrug -924, was 1,9 % der Gesamtsterblichkeit 2019 in Rheinland-Pfalz entspricht.

Im Monat der Flutkatastrophe im Ahrtal (07/2021) betrug bei lediglich einem gemeldeten COVID-19-Todesfall die modellierte Übersterblichkeit 196 (95 %-PI [151; 241]) in den Kreisen Ahrweiler (122), Altenkirchen (26), Koblenz (25) und Bitburg-Prüm (23). Unmittelbar durch die Flutkatastrophe verstarben 133 Personen in Rheinland-Pfalz [31].

## Diskussion

Die Zahl der COVID-19-attribuierten Sterbefälle in Deutschland variierte von 01/2020 bis 07/2021 erheblich. Konsistent mit früheren Veröffentlichungen zur Übersterblichkeit in Deutschland [6, 7] zeigte sich in der Mehrheit der Monate keine Evidenz für eine Übersterblichkeit in den Bundesländern bzw. in den Kreisen von Rheinland-Pfalz. In den meisten, aber nicht allen Bundesländern sowie Kreisen von Rheinland-Pfalz wiesen die Sterbefälle deutliche zeitliche Trends auf, die mit der ersten (03 bis 04/2020) und zweiten Welle (11/2020 bis 01/2021) der Pandemie zusammenfielen [32].

Maßnahmen, die von den Bundesländern zur Pandemieeindämmung ergriffen wurden, etwa die Schließung von Geschäften oder Schulen, das Verbot großer Menschenansammlungen und Reisebeschränkungen, waren nicht immer identisch und wurden nicht gleichzeitig erlassen [33]. Diese Maßnahmen wurden jedoch meist über die Bundesländer hinweg koordiniert, wichtige Richtlinien wurden zudem zentral von der Bundesregierung erlassen. Die Heterogenität der Übersterblichkeit auf Ebene der Bundesländer erscheint damit deutlich größer als die Heterogenität der gesundheitspolitischen Interventionen [33]. Erhebliche Unterschiede in der Übersterblichkeit zeigten sich auch zwischen Kreisen in Rheinland-Pfalz, obwohl anzunehmen ist, dass die Interventionen innerhalb eines Bundeslandes homogener waren als zwischen den Bundesländern.

In den meisten Regionen konnte eine hohe absolute Übereinstimmung zwischen Übersterblichkeit und COVID-19-attribuierter Mortalität festgestellt werden. Im Spätsommer und Herbst 2020 wiesen jedoch einige Bundesländer und Kreise Übersterblichkeiten auf, die höher als die COVID-19-attribuierten Sterbefälle waren, so z. B. in Hamburg und im Saarland sowie in geringerem Maße auch in Bayern, Bremen und Hessen. Die vorhergesagten Sterbefälle in Hamburg und im Saarland waren in der Sensitivitätsanalyse für 2019 durchweg zu niedrig (siehe Onlinematerial), nicht aber in Bayern, Bremen und Hessen. Indirekte Effekte der Pandemie auf die Mortalität können als Erklärung für die Diskrepanz in diesen Fällen nicht ausgeschlossen werden.

Auch in Sachsen und Sachsen-Anhalt lag die Übersterblichkeit im Winter 2020/2021 über der COVID-19-attribuierten Sterblichkeit, die zu diesem Zeitpunkt sehr hoch war. Für beide Bundesländer kommt als Erklärung neben indirekten Effekten der Pandemie auch eine Untererfassung von COVID-19-verursachten Sterbefällen in Betracht, etwa bedingt durch zu geringe Testkapazitäten.

Dagegen lag die Übersterblichkeit in vielen Bundesländern bzw. Kreisen in Rheinland-Pfalz im Frühjahr 2021 unter der COVID-19-assoziierten Sterblichkeit und war z. T. deutlich negativ. Diese unerwartet geringe Sterblichkeit schloss sich direkt an die Übersterblichkeit im Winter 2020/2021 an, die mit einer hohen COVID-19-attribuierten Sterblichkeit zusammenfiel. Eine mögliche Erklärung ist der Ausgleich einer COVID-19-bedingten kurzzeitigen Vorverlagerung von Sterbefällen (*Harvesting*-Effekt). Als weitere Erklärung ist die Möglichkeit zu berücksichtigen, dass die getroffenen Maßnahmen zur Pandemieeindämmung auch vor anderen Infektionskrankheiten schützten und assoziierte Sterbefälle verhinderten. Konsistent damit ist, dass im Winter 2020/2021 keine Influenzaaktivität (definierte Grippewelle) gemeldet wurde [22].

Eine frühere Analyse der Übersterblichkeit in Deutschland von Kalenderwoche 10 bis 22 des Jahres 2020 [4] zeigte keine signifikant erhöhte Sterblichkeit im Vergleich zur durchschnittlichen Sterblichkeit der Jahre 2016 bis 2019, wobei der demografische Wandel berücksichtigt wurde. Eine Analyse bis Kalenderwoche 48 des Jahres 2020 zeigte während der ersten und zweiten Pandemiewelle eine Übersterblichkeit nur in der Altersgruppe ab 80 Jahren [7]. De Nicola et al. [6] stellten alternative Berechnungsmethoden der für 2020 erwarteten wöchentlichen Sterblichkeit in Gesamtdeutschland auf Basis von Sterbetafeln mit einer feineren Altersstratifizierung vor. Die resultierenden Schätzungen der Übersterblichkeit sind mit unseren Ergebnissen kompatibel. Unsere Analyse unterscheidet sich von diesen Publikationen u. a. durch die höhere räumliche Auflösung mit bundesland- bzw. kreisspezifischer Betrachtung sowie durch die Berücksichtigung eines langfristigen Trends der Sterblichkeitsrate und des Einflusses der Lufttemperatur. Sowohl der ungewöhnlich milde Winter zu Beginn des Jahres 2020 als auch die Fortsetzung des leicht abnehmenden Trends der Sterblichkeit [8] senkten in unserer Analyse die erwartete Sterblichkeit und erhöhten somit die Übersterblichkeit.

Die Übersterblichkeit stimmte mit der beobachteten Heterogenität der COVID-19-attribuierten Mortalität weitgehend überein, sowohl in Bezug auf zeitliche Trends wie geografische Muster. Diese Übereinstimmung ist kompatibel mit der Annahme, dass die Übersterblichkeit weitgehend durch die COVID-19-attribuierte Sterblichkeit erklärt wird.

Die ausgeprägte geografische und zeitliche Heterogenität der Sterberaten, sowohl auf Ebene der Bundesländer als auch auf Kreisebene in Rheinland-Pfalz, verdeutlicht, dass aggregierte Kennzahlen, die sich aus der Integration von Daten über große Regionen und lange Zeiträume ergeben, wenig aussagekräftig für die Charakterisierung der Auswirkungen der COVID-19-Pandemie auf die öffentliche Gesundheit sind.

### Stärken und Limitationen

Durch die Berücksichtigung der Bevölkerungsstruktur und anderer starker Einflüsse auf die Sterblichkeit liefert diese Studie robuste Schätzungen der Übersterblichkeit während der COVID-19-Pandemie. Die Studie verbessert damit Schätzungen, die allein auf dem historischen Vergleich der durchschnittlichen absoluten Sterbefallzahlen der letzten Jahre beruhen oder nur die Altersstruktur berücksichtigen. Mit unseren Methoden geschätzte Übersterblichkeiten können valide zwischen Ländern mit unterschiedlichen demografischen Strukturen verglichen werden, was internationale Studien ermöglicht.

Diese Studie hat mehrere Limitationen. Die Bevölkerungsdaten der Bundesländer von 2021 stammen aus einer Vorausberechnung, die zugehörigen Sterbedaten waren vorläufig. Das Statistische Bundesamt schätzt jedoch, dass die Sterberegistrierungen im Durchschnitt nach 4 Wochen zu 97 % vollständig sind [16]. Während die Sterbefälle nach Möglichkeit dem Wohnort zugeordnet wurden, wurde bei unbekanntem Wohnort der Ort der Meldung imputiert. Eine fein aufgelöste Altersadjustierung bis zum Alter von 65 Jahren war auf Ebene der Bundesländer nicht möglich. Für die Altersgruppe ab 65 Jahren mit weit höheren Sterberaten lagen demgegenüber feiner stratifizierte Daten vor.

Die Daten für COVID-19-attribuierte Todesfälle auf Kreisebene in Rheinland-Pfalz wiesen zeitliche Unsicherheiten auf, da z. T. das möglicherweise vom Sterbedatum abweichende Meldedatum verwendet wurde. Der Ort der COVID-19-attribuierten Todesfälle wurde entsprechend der meldenden Einrichtung festgelegt, die sich vom Wohnort unterscheiden konnte.

Die Wahl von 2015 bis 2019 als historischer Vergleichszeitraum für die Sterblichkeitsraten von 01/2020 bis 07/2021 wurde nicht auf Grundlage theoretischer Überlegungen, sondern auf Basis der Datenverfügbarkeit getroffen. Während jedoch die Lebenserwartung zwischen 1990 und 2010 erheblich gestiegen ist, waren die altersstandardisierten Sterberaten von 2015 bis 2019 stabiler [8]. Durch die Adjustierung der modellierten Sterblichkeitsraten für Lufttemperatur und Influenzaaktivität wurden außerdem Mortalitätsspitzen während der schweren Influenzawelle im Frühjahr 2018 oder während sommerlicher Hitzewellen in den Jahren 2018 und 2019 bei der Modellierung der erwarteten Sterblichkeit berücksichtigt.

Die vorliegende Analyse modelliert keine zeitlichen Mortalitätsverschiebungen. Solche Effekte können Anfang 2020 aufgetreten sein, als in einem ungewöhnlich warmen Winter eine gleichzeitige deutliche Untersterblichkeit zu beobachten war. Einige Todesfälle könnten dadurch leicht verzögert worden und dann mit der ersten Welle der COVID-19-Pandemie zusammengefallen sein. Dies hätte eine nach oben verzerrte Schätzung der Übersterblichkeit zur Folge.

Die vorgestellte Analyse geht davon aus, dass die durch COVID-19 verursachte Sterblichkeit und die durch indirekte Folgen der Pandemie verursachte Sterblichkeit keine Schnittmenge haben. Ein Moderationseffekt, bei dem z. B. eine beeinträchtigte Gesundheitsversorgung zu einer höheren Anfälligkeit für einen schweren COVID-19-Verlauf führt, wäre nicht sichtbar.

Ebenso wäre eine falsche Klassifikation anderer Todesursachen als COVID-19-Todesfall von tatsächlichen COVID-19-Todesfällen nicht zu unterscheiden. Todesbescheinigungen aus dem Jahr 2020 wurden vorläufig durch das Statistische Bundesamt [34] und das Statistische Landesamt Rheinland-Pfalz [35] hinsichtlich der angegebenen Todesursache (Grundleiden) und der Begleiterkrankungen ausgewertet. Die Ergebnisse zeigen, dass in 83,0 % (Deutschland) bzw. 81,5 % (Rheinland-Pfalz) der Todesbescheinigungen, die COVID-19 als Erkrankung aufführten, COVID-19 als Todesursache angegeben war. In den verbleibenden 17,0 % bzw. 18,5 % wurde COVID-19 als Begleiterkrankung genannt. Aufgrund anderer Meldewege und einer anderen Methodik der Zuschreibung von COVID-19 zu einem Todesfall sind diese Ergebnisse nicht unmittelbar auf die gemäß IfSG an das RKI gemeldeten Daten übertragbar [34]. Zudem kann die Zuordnung von Grundleiden und Begleiterkrankung nicht immer das Zusammenwirken von am Todesfall beteiligten Ursachen mit unterschiedlich starken Beiträgen zur Lebenszeitverkürzung vollständig widerspiegeln.

Würde man annehmen, dass die in dieser Auswertung verwendeten Daten für COVID-19-Todesfälle knapp 20 % zu hoch wären, wäre vor allem die Höhe der Übersterblichkeit von 11/2020 bis 01/2021 in vielen Bundesländern stärker erklärungsbedürftig.

Detaillierte Analysen der zeitlichen Entwicklung des relativen Anteils einzelner Todesursachen sind erforderlich, um langfristige indirekte Effekte auf die Mortalität zu erfassen, die durch Unterbrechungen der Gesundheitsversorgung und geringere Inanspruchnahme medizinischer Hilfe verursacht wird. Eine solche Analyse würde jedoch durch Unsicherheiten bei der Todesursachencodierung beeinflusst und müsste konkurrierende Risiken gegenüber dem Tod durch COVID-19 berücksichtigen.

### Schlussfolgerungen

Die Analyse der Gesamtmortalität unter Berücksichtigung der Altersstruktur der Bevölkerung und anderer systematischer Einflüsse auf die Mortalität ermöglicht robuste Vergleiche der zeitlichen und regionalen Auswirkungen der COVID-19-Pandemie auf die öffentliche Gesundheit.

Zwischen der Übersterblichkeit und den COVID-19-attribuierten Sterbefällen bestand sowohl über die Regionen als auch über die Zeit hinweg ein enger Zusammenhang. Dies legt nahe, dass die COVID-19-attribuierte Sterblichkeit die Hauptquelle der zeitlich und räumlich begrenzt auftretenden Übersterblichkeit von 01/2020 bis 07/2021 in Deutschland war. Es zeigten sich kaum Hinweise auf größere Beiträge indirekt verursachter Sterblichkeit durch gesundheitspolitische Maßnahmen zur Pandemieeindämmung.

Die während der Sommermonate 2020 in einigen Bundesländern beobachtete Übersterblichkeit trotz weniger COVID-19-attribuierter Sterbefälle könnte jedoch auf indirekt verursachte Sterblichkeit von geringerem, aber dennoch relevantem Ausmaß hindeuten.

## Supplementary Information




