# Supplementary material for: Explaining the age-adjusted excess mortality with COVID-19-attributed deaths from January 2020 to July 2021
Source: Bundesgesundheitsblatt Gesundheitsforschung Gesundheitsschutz. 2021 Dec 4;65(3):378–87. [Article in German] doi: 10.1007/s00103-021-03465-z (PMC8643124; doi:10.1007/s00103-021-03465-z)
Supplement: Supplementary file 1 [file 103_2021_3465_MOESM1_ESM.pdf]

Bundesgesundheitsblatt – Gesundheitsforschung – Gesundheitsschutz 3/2022

Onlinematerial zum Beitrag

Erklärbarkeit der altersadjustierten Übersterblichkeit mit den COVID-19-attribuierten Sterbefällen von Januar 2020 bis Juli 2021

Daniel Wollschläger[1], Irene Schmidtmann[1], Sebastian Fückel[2], Maria Blettner[1], Emilio Gianicolo[1,3]

[1] Institut für Medizinische Biometrie, Epidemiologie und Informatik (IMBEI), Universitätsmedizin der Johannes Gutenberg-Universität Mainz, Mainz, Deutschland

[2] Statistisches Landesamt Rheinland-Pfalz, Bad Ems, Deutschland

[3] Institute of Clinical Physiology of the Italian National Research Council (IFC-CNR), Lecce, Italien

Korrespondenzadresse:

PD Dr. Daniel Wollschläger

Institut für Medizinische Biometrie, Epidemiologie und Informatik (IMBEI)

Universitätsmedizin der Johannes Gutenberg-Universität Mainz

Langenbeckstraße 1

55131 Mainz

wollschlaeger@uni-mainz.de

Inhalt:

Material und Methoden: Datenquellen, Regressionsmodell

Sensitivitätsanalysen: Anpassung Regression mit Maximum Likelihood, Modell für 2019

Glossar

Tabellen: Geschätzte Koeffizienten Regressionsmodelle, Übersterblichkeit in Bundesländern und Kreisen von Rheinland-Pfalz

1 Material und Methoden

1.1 Datenquellen

1.1.1 Bevölkerungszahlen Rheinland-Pfalz

Das Statistische Landesamt Rheinland-Pfalz stellte Bevölkerungsdaten stratifiziert nach Kreis, Geschlecht und Altersgruppe (10 Jahre) zur Verfügung. Für 2014 bis 2019 lagen die Daten als geprüfte Jahreszahlen zum Stichtag 31. Dezember vor. Für 2019 bis 2021 lagen vorläufige monatliche Zählungen aus der Gemeindestatistik vor (KommWis - Gesellschaft für Kommunikation und Wissenstransfer mbH, Mainz).

Da die Registrierung von Einwohnerinnen und Einwohnern mit einer unbekannten Verzögerung erfolgt, sind die monatlichen Zahlen mit Unsicherheit behaftet. Für 2019 lagen die Bevölkerungsdaten sowohl als geprüfte Zahlen zum Jahresende als auch als vorläufige monatliche Zahlen vor. Mithilfe eines Poisson-Regressionsmodells wurden die vorläufigen monatlichen Zahlen für 2019 mit Hilfe der linear interpolierten Zahlen am Jahresende kalibriert. Kovariate war die Altersgruppe, den Offset bildeten die logarithmierten vorläufigen monatliche Zahlen. Die Vorhersagen aus diesem Regressionsmodell wurden als geschätzte monatliche Bevölkerungszahlen für 2019 bis 2021 in allen Analysen verwendet.

1.1.2 Sterbefälle

Die Sterbefälle aus den Bundesländern für die Jahre 2015 bis 2021 wurden vom Statistischen Bundesamt als monatliche Zahlen stratifiziert nach Altersgruppe (0-64, 65-74, 75-84, 85+ Jahre) und Geschlecht zur Verfügung gestellt [1]. Für die Jahre 2020 und 2021 waren die Daten vorläufig, Informationen über Sterbefälle außerhalb des Bundeslandes, in dem der Wohnsitz war, waren noch nicht mit anderen Bundesländern ausgetauscht worden.

Das Statistische Landesamt Rheinland-Pfalz lieferte für die Jahre 2015 bis 2019 vollständig verifizierte Sterbefälle stratifiziert nach Landkreis, Geschlecht und Altersgruppe (10 Jahre). Für die Jahre 2020-2021 wurden vorläufige Sterbefälle von den Standesämtern zur Verfügung gestellt und danach einer ersten Qualitätskontrolle unterzogen. Verspätete Korrekturen durch Standesämter sowie der Austausch von Informationen über Sterbefälle außerhalb von Rheinland-Pfalz waren noch nicht durchgeführt worden.

1.1.3 Regionaler sozioökonomischer Deprivationsindex

Als Maß für die regionale sozioökonomische Deprivation wurde der kreisspezifische *German Index of Socioeconomic Deprivation* (GISD) 2014 [2,3] auf Basis von Indikatoren für 2015 bis 2017 verwendet. Für die Jahre 2018 bis 2021 wurde der entsprechende Wert des Jahres 2017 fortgeschrieben. Der Index für jedes Bundesland wurde als bevölkerungsgewichtetes Mittel der entsprechenden kreisspezifischen Werte berechnet.

1.1.4 Temperaturdaten Rheinland-Pfalz

Die Agrarmeteorologie Rheinland-Pfalz stellte die Temperaturdaten in Form täglicher Mittelwerte jeweils aller von ihr sowie vom Deutschen Wetterdienst in Rheinland-Pfalz unterhaltenen Messstationen zur Verfügung. Die Berechnung der kreisspezifischen monatlichen Mittelwerte erfolgte durch Mittelung über alle im jeweiligen Kreis befindlichen Stationen. Da sich im Landkreis Zweibrücken keine Messstation befand, wurden die Temperaturen mit den Durchschnittstemperaturen der übrigen Landesregion Pfalz imputiert, zu der neben Zweibrücken die Kreise Südwestpfalz, Kusel und Kaiserslautern zählen.

1.1.5 Saisonale Influenza-Aktivität

Die Daten zur Aktivität der saisonalen Influenza wurden vom Robert Koch-Institut [4] als wöchentliche kreisspezifische Werte bereitgestellt. Diese Werte wurden teilweise aus Meldungen niedergelassener Ärztinnen und Ärzte abgeleitet und können deshalb an Feiertagen, an denen Arztpraxen geschlossen waren, nach unten verzerrt sein. Für Weihnachten und Ostern wurde dieser Effekt korrigiert, indem zwischen benachbarten Wochen linear interpoliert wurde, wenn die Aktivität in der Kalenderwoche des Feiertags niedriger war als sowohl die Aktivität in der vorangehenden als auch in der folgenden Kalenderwoche. Zur Ableitung der monatlichen saisonalen Influenza-Aktivität wurde jede Kalenderwoche dem Monat des entsprechenden Mittwochs zugeordnet.

1.1.6 COVID-19-attribuierte Sterbefälle

Vom Robert Koch-Institut wurden zwei Datensätze zur COVID-19-attributierten Mortalität zur Verfügung gestellt. Die für die Analyse auf Ebene der Bundesländer verwendeten Daten wurden bereits in einer früheren Auswertung des Statistischen Bundesamtes [1] verwendet. In diesem Datensatz wurde die wöchentliche Anzahl der verstorbenen COVID-19-Fälle (Meldungen gemäß Infektionsschutzgesetz) nach dem Sterbedatum erfasst [5]. Zur Ableitung der monatlichen Sterbefälle wurde jede Kalenderwoche dem Monat des entsprechenden Mittwochs zugeordnet.

Wöchentliche bundeslandspezifische COVID-19-attributierte Sterbefälle mit einer Häufigkeit von 1 bis 3 wurden dabei nur in der Form “<4” angegeben. Um diese Werte zu imputieren, wurde die Differenz zwischen der Summe aller genau angegebenen bundeslandspezifischen Werte und der Gesamtzahl für Deutschland für dieselbe Woche gleichmäßig auf die Bundesländer mit “<4”-Angaben verteilt. In der Kalenderwoche

10/2020 wurde die deutsche Gesamtzahl ebenfalls als “<4” erfasst. In diesem Fall wurden die zwei bundeslandspezifischen “<4”-Angaben jeweils auf 1 gesetzt.

Die für die Analyse auf Kreisebene in Rheinland-Pfalz verwendeten COVID-19-attribuierten Sterbefälle sind eine Teilmenge der Meldungen gemäß Infektionsschutzgesetz, die im COVID-19-Dashboard des Robert Koch-Instituts [6] dargestellt sind. COVID-19-attributierte Sterbefälle wurden nach dem Meldedatum registriert, wenn kein Sterbedatum vorhanden war [7].

1.2 Regressionsmodell für Mortalitätsraten

Die Negativ-Binomial Regressionsmodelle wurden mit der Hamilton Monte Carlo-Methode angepasst, wie sie im Paket brms [8] für die Bayes’sche Modellierungssprache Stan [9] implementiert ist. Mit μ als Erwartungswert der Sterbefälle in einer Poisson-Zelle, β_0 als Rate der Sterbefälle, wenn alle stetigen Kovariaten gleich 0 und alle kategorialen Kovariaten gleich der Referenzkategorie sind sowie den weiteren Parametern β als Koeffizienten der Prädiktoren lautet die Modellgleichung:

$$\begin{aligned}
\ln\mu \quad = \quad & \beta_0 + \\
& \beta_1 \mathrm{GISD} + \\
& \beta_2 \mathrm{Temp}_{<18} + \beta_3 \mathrm{Temp}_{>18} + \\
& \beta_4 \mathrm{Datum\ numerisch} + \beta_5 \sin\left(\mathrm{Datum}_{[0,2\pi]}\right) + \beta_6 \cos\left(\mathrm{Datum}_{[0,2\pi]}\right) \\
& \beta_7 \mathrm{Influenza} + \\
& \beta_8 \mathrm{Geschlecht}_{\mathrm{männlich}} + \\
& \beta_9 \mathrm{Alter}_{\mathrm{Gruppe\ 2}} + \beta_{\dots} \mathrm{Alter}_{\mathrm{Gruppe\ \dots}} + \\
& \beta_{\dots} \mathrm{Geschlecht}_{\mathrm{männlich}} \times \mathrm{Alter}_{\mathrm{Gruppe\ 2}} + \\
& \beta_{\dots} \mathrm{Geschlecht}_{\mathrm{männlich}} \times \mathrm{Alter}_{\mathrm{Gruppe\ \dots}} + \\
& \ln \mathrm{Personenjahre}
\end{aligned}$$

$\mathrm{Temp}_{<18}$ und $\mathrm{Temp}_{>18}$ stehen für die beiden stückweise linearen Spline-Terme mit innerem Knoten bei 18°C. $\mathrm{Datum}_{[0,2\pi]}$ steht für das auf das Intervall $[0,2\pi]$ reskalierte numerische Datum. Im Modell auf Ebene der Bundesländer sind die Altersgruppen 0-64

Jahre (Referenzgruppe), 65-74 Jahre, 75-84 Jahre, 85+ Jahre. Im Modell für die Kreise in Rheinland-Pfalz sind die Altersgruppen 0-9 Jahre, 10-19 Jahre, 20-29 Jahre, 30-39 Jahre (Referenzgruppe), 40-49 Jahre, 50-59 Jahre, 60-69 Jahre, 70-79 Jahre, 80-89 Jahre, 90+ Jahre.

Für β_0 wurde als Prior eine Normalverteilung mit Mittelwert 0 und Standardabweichung 15 verwendet. Für die übrigen Regressionsparameter wurde als Prior jeweils eine Normalverteilung mit Mittelwert 0 und Standardabweichung 5 verwendet. Aus 20 Markov-Ketten mit jeweils einer Aufwärmphase von 5.000 Ziehungen, gefolgt von 5.000 Ziehungen mit einem Ausdünnungsintervall von 5 wurden insgesamt 20.000 Ziehungen pro Modell beibehalten.

Die Qualität des Monte-Carlo-Samplings wurde anhand diagnostischer Diagramme für die Autokorrelation in jeder Markov-Kette sowie für die Mischung der Ketten bzgl. jedes Parameterschätzers bewertet. Es gab keine Parameter mit einem effektiven Stichprobenumfang von weniger als 80% des gesamten Stichprobenumfangs oder mit einem $\hat{R}$-Wert über 1,01.

2 Sensitivitätsanalysen

2.1 Anpassung der Regression mit Maximum-Likelihood-Methode

2.1.1 Bundesländer Deutschland

Referenzkategorie für den Faktor Altersgruppe: 0-64 Jahre. Referenzkategorie für den Faktor Geschlecht: weiblich.

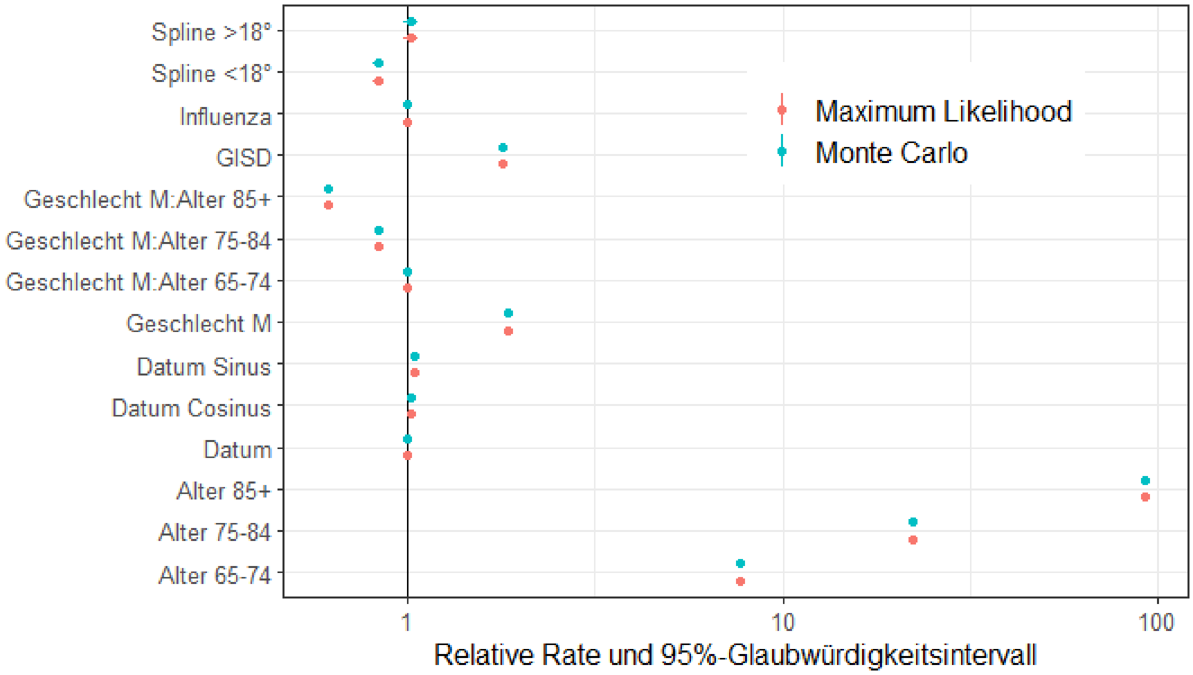

Abbildung Z1: Geschätzte relative Raten (RR) mit 95%-Glaubwürdigkeitsintervallen (Monte-Carlo-Fit) und 95%-Konfidenzintervallen (Maximum-Likelihood-Fit) für das Negativ-Binomial-Regressionsmodell für Mortalitätsraten von 2015 bis 2019 in Deutschland.

2.1.2 Kreise in Rheinland-Pfalz

Referenzkategorie für den Faktor Altersgruppe: 30-39 Jahre. Referenzkategorie für den Faktor Geschlecht: weiblich.

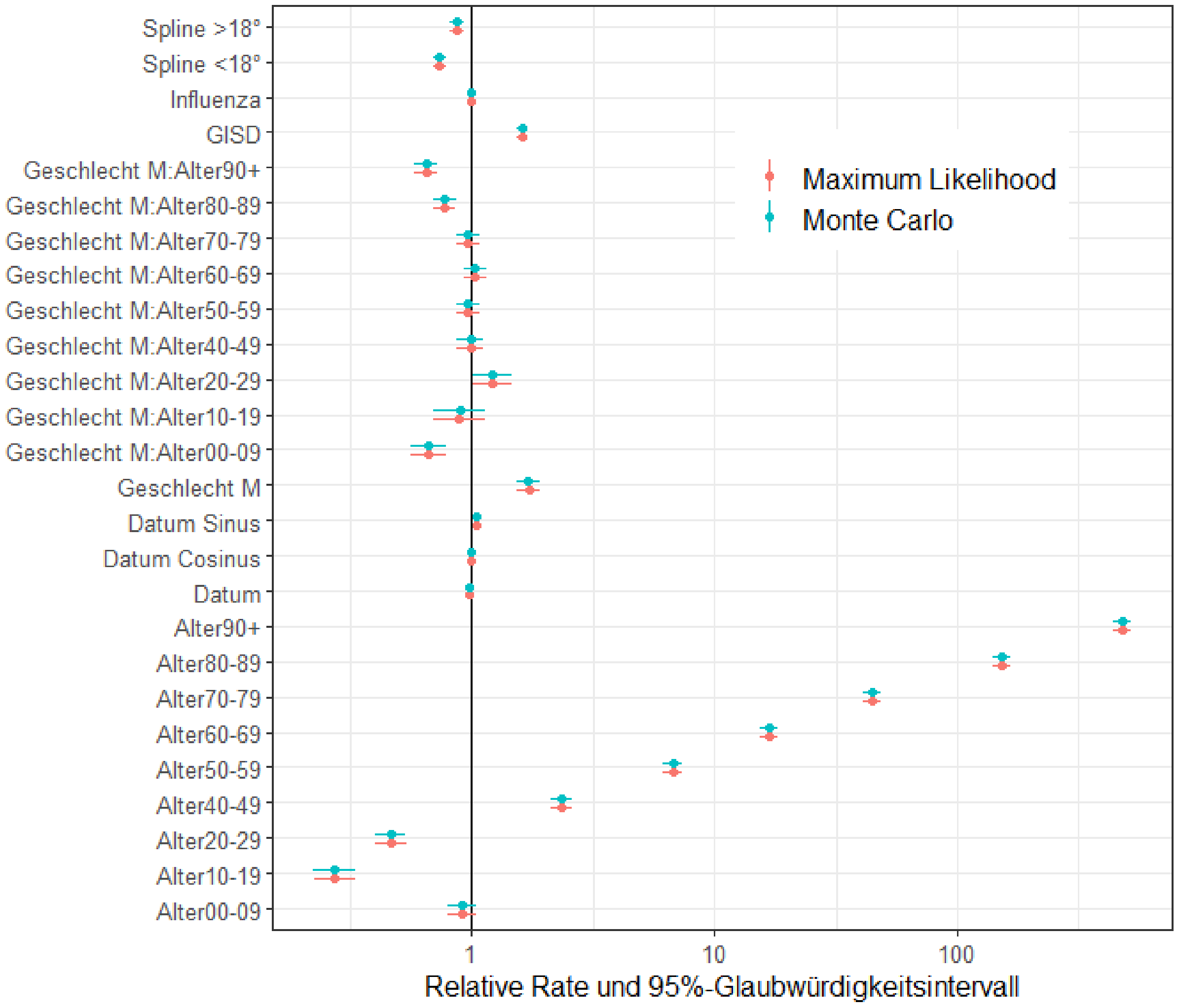

Abbildung Z2: Geschätzte relative Raten (RR) mit 95%-Glaubwürdigkeitsintervallen (Monte-Carlo-Fit) und 95%-Konfidenzintervallen (Maximum-Likelihood-Fit) für das Negativ-Binomial-Regressionsmodell für Mortalitätsdaten von 2015 bis 2019 in Rheinland-Pfalz.

2.2 Vergleich zwischen beobachteten und vorhergesagten Sterbefällen im Jahr 2019

Um zu überprüfen, ob die gewählte Regressionsmodellierung zu unverzerrten Schätzungen der zu erwartenden Sterbefälle in Abwesenheit einer Pandemie führt, wurden die Regressionsmodelle auch an Daten von 2015 bis 2018 angepasst, um die beobachtete und vorhergesagte Sterblichkeit im Jahr 2019 vergleichen zu können.

2.2.1 Bundesländer in Deutschland

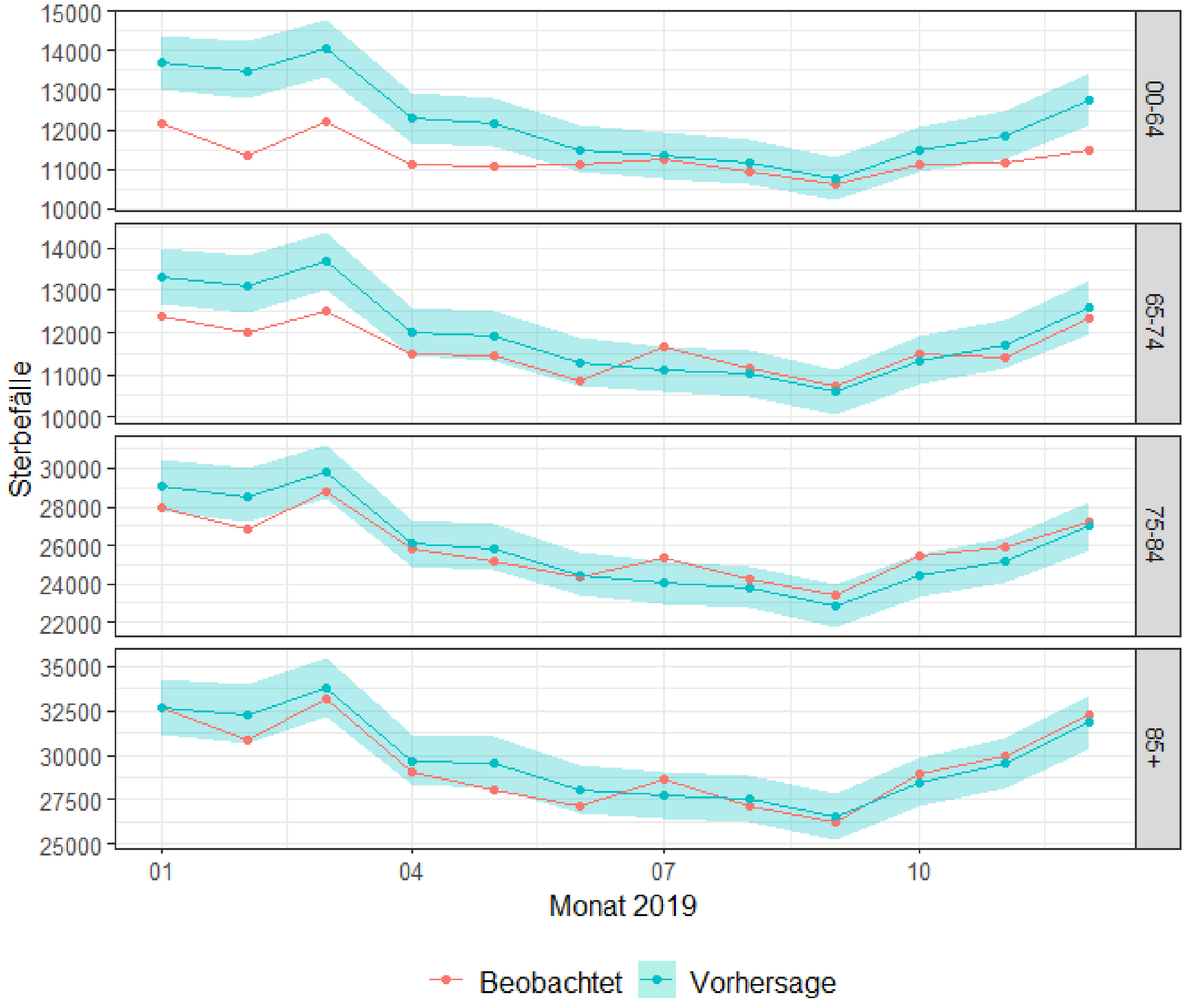

Abbildung Z3: Beobachtete und vorhergesagte Sterbefälle in Deutschland im Jahr 2019, stratifiziert nach Altersgruppen, basierend auf einem Negativ-Binomial-Regressionsmodell, das an Daten aus den Jahren 2015 bis 2018 angepasst wurde, einschließlich 95%-

Vorhersageintervalle für die vorhergesagten Sterbefälle.

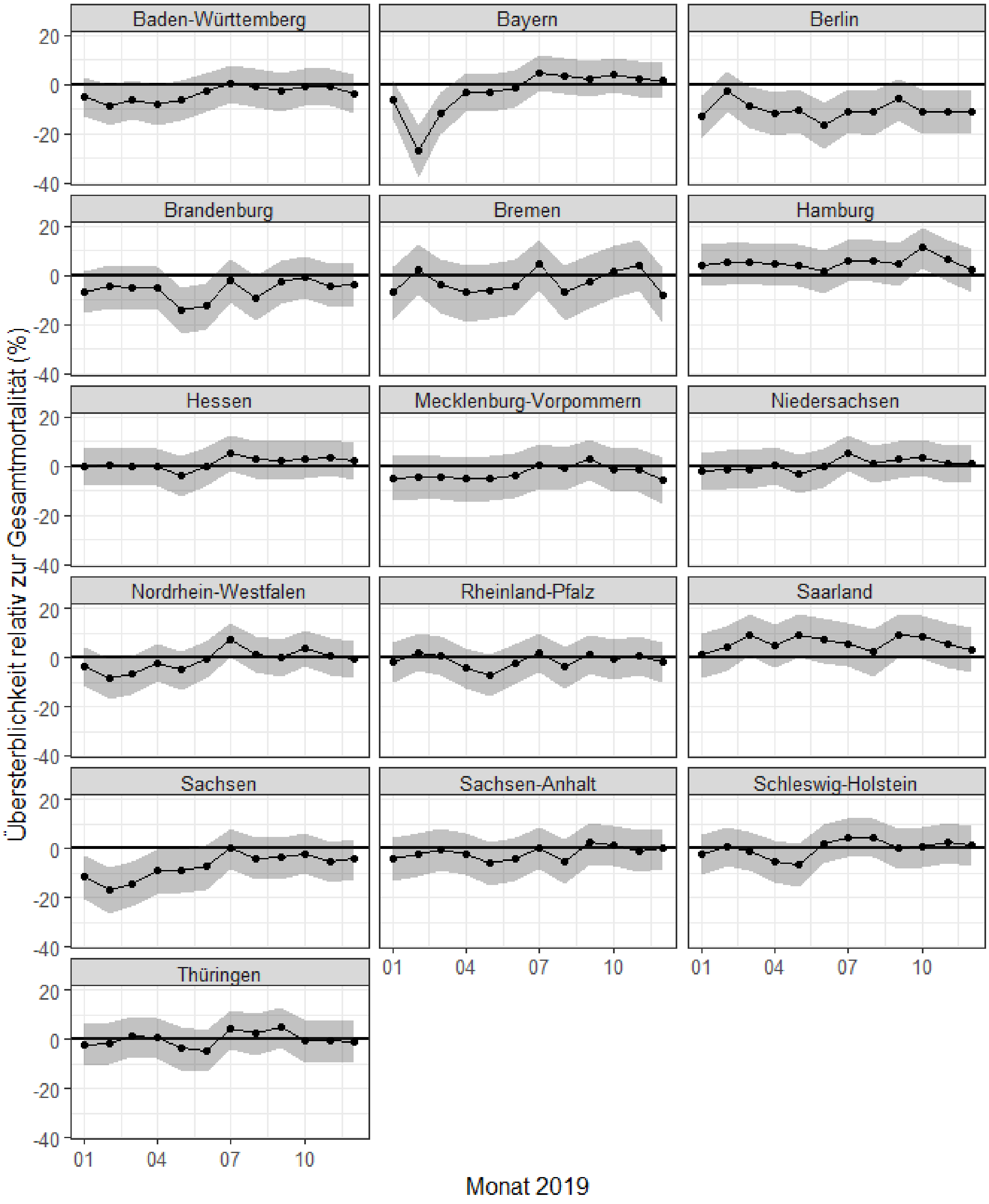

Abbildung Z4: Übersterblichkeit relativ zur Gesamtmortalität in Deutschland im Jahr 2019, stratifiziert nach Bundesland, basierend auf einem Negativ-Binomial-Regressionsmodell, das an Daten aus den Jahren 2015 bis 2018 angepasst wurde, einschließlich 95%-Vorhersageintervalle.

2.2.2 Kreise in Rheinland-Pfalz

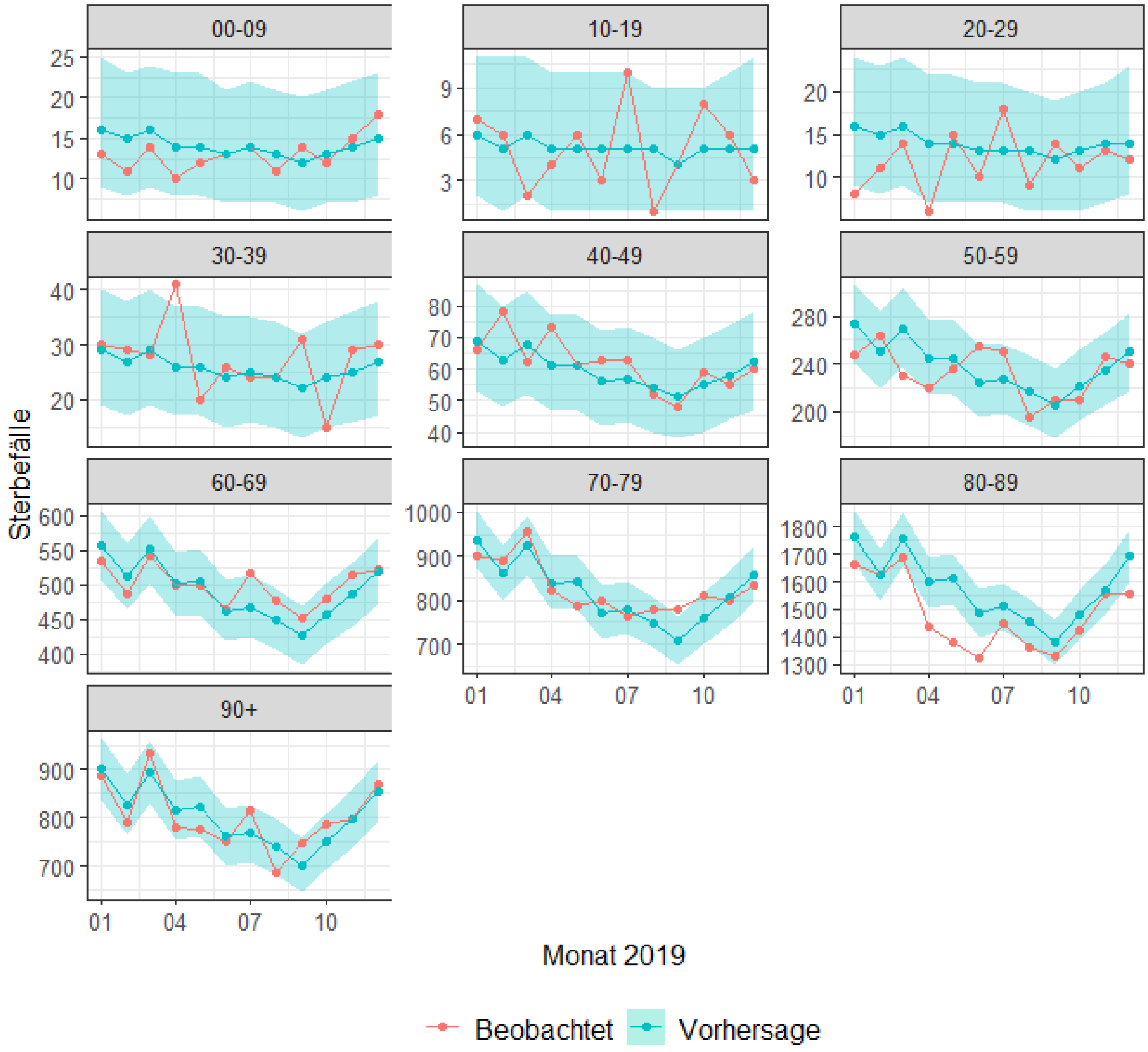

Abbildung Z5: Beobachtete und vorhergesagte Sterbefälle in Rheinland-Pfalz im Jahr 2019, stratifiziert nach Altersgruppen, basierend auf einem Negativ-Binomial-Regressionsmodell, das an Daten aus den Jahren 2015 bis 2018 angepasst wurde, einschließlich 95%-Vorhersageintervalle für die vorhergesagten Sterbefälle.

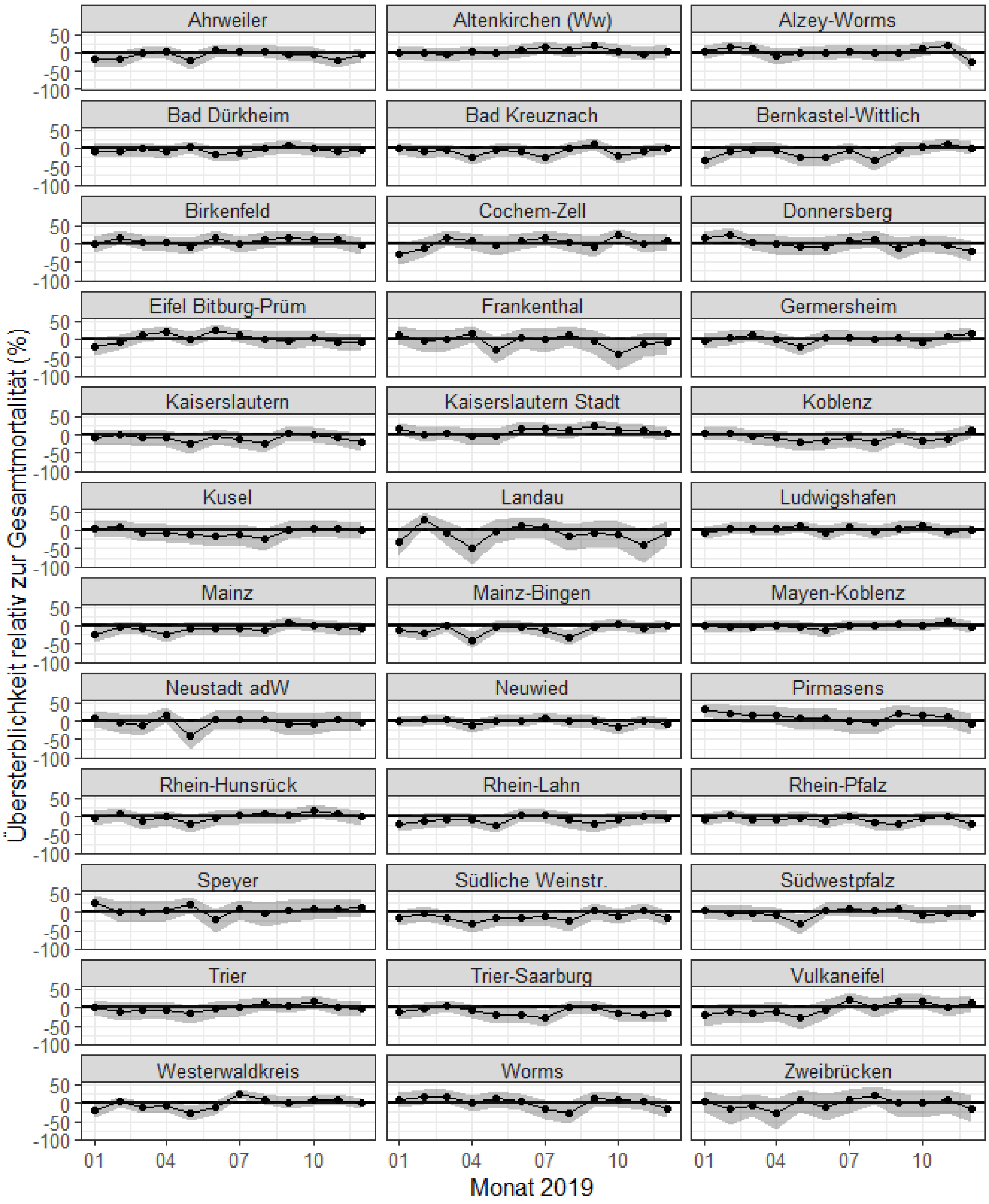

Abbildung Z6: Übersterblichkeit relativ zur Gesamtmortalität in Rheinland-Pfalz im Jahr 2019, stratifiziert nach Kreis, basierend auf einem Negativ-Binomial-Regressionsmodell, das an Daten aus den Jahren 2015 bis 2018 angepasst wurde, einschließlich 95%-Vorhersageintervalle.

3 Glossar

Mortalitätsrate

Anzahl der Sterbefälle pro Zeiteinheit.

Negativ-Binomial-Regressionsmodell

Statistisches Modell ähnlich der Poisson-Regression, in dem die logarithmierte Ereignisrate als Zielgröße linear modelliert wird. Im Unterschied zur Poisson-Regression wird zugelassen, dass die Varianz der Häufigkeiten größer als ihr Erwartungswert ist.

Poisson-Zelle

Beobachtungseinheit des Negativ-Binomial-Regressionsmodells. Pro Zelle geht jeweils ein Wert für den betrachteten Endpunkt (Ereignishäufigkeit) sowie für jede Kovariate in das Modell ein. Die Poisson-Zellen werden aus der vollständigen Kreuzklassifikation der kategorialen Kovariaten gebildet.

Offset

Der exponenzierte *Offset* ist der Faktor, mit dem die im Regressionsmodell modellierte Ereignisrate multipliziert wird, um die in einer Poisson-Zelle beobachtete Häufigkeit zu erhalten. Der Offset ist gleich der logarithmierten *Personenzeit* in einer Poisson-Zelle. Die Personenzeit ist die gesamte Zeit, die alle in einer Poisson-Zelle erfassten Personen in diese Zelle einbringen.

Stückweise linearer Spline

Eine in Segmenten abschnittsweise lineare Funktion mit potentiell unterschiedlicher Steigung je Segment. Im gewählten Modell wurde zusätzlich festgelegt, dass sich die

Segmente am Bruchpunkt (*Knoten*) zwischen ihnen berühren, die Funktion also kontinuierlich ist.

Posterior-Verteilung

Im Monte-Carlo-Verfahren auf Basis von simulierten Zufallsprozessen ermittelte statistische Verteilung der vorhergesagten Häufigkeit. In die Verteilung fließt sowohl die Unsicherheit in der Schätzung der Parameter des Regressionsmodells für den Erwartungswert ein als auch die Varianz der beobachteten Häufigkeiten bei gegebenem Erwartungswert.

BCa Bootstrap-Konfidenzintervall

Verfahren zur Bestimmung des Konfidenzintervalls für einen statistischen Parameter auf Basis von *Bootstrapping*.
Bootstrapping: *Resampling* Verfahren, das die Verteilung des Parameterschätzers nicht parametrisch berechnet sondern aus mehrfachen mit Zurücklegen gebildeten Ziehungen der empirisch beobachteten Werte bildet.
BCa (*Bias corrected and accelerated*) - Verfahren zur Berechnung des Konfidenzintervalls, das bei schiefen Verteilungen sicherstellt, dass die tatsächliche Abdeckungswahrscheinlichkeit der nominellen (95%) entspricht.

4 Tabellen

Tabelle Z1: Geschätzte relative Mortalitätsraten (RR) und 95%-Unsicherheitsintervalle (RR 2,5%, RR 97,5%) für das Negativ-Binomial-Regressionsmodell für Mortalitätsdaten von 2015 bis 2019 in Deutschland. GISD: *German Index of Socioeconomic Deprivation*

Koeffizient	RR	RR 2,5%	RR 97,5%
Datum Sinus	1,04	1,03	1,04
Datum Cosinus	1,02	1,00	1,03
GISD	1,80	1,75	1,84
Spline <18°	0,83	0,80	0,86
Spline >18°	1,02	0,97	1,07
Datum	0,99	0,99	1,00
Influenza	1,00	1,00	1,00
Geschlecht M	1,85	1,83	1,87
Alter 65-74	7,67	7,59	7,76
Alter 75-84	22,36	22,13	22,59
Alter 85+	93,26	92,31	94,20
Geschlecht M: Alter 65-74	1,00	0,99	1,02
Geschlecht M: Alter 75-84	0,83	0,82	0,84
Geschlecht M: Alter 85+	0,61	0,60	0,62

Tabelle Z2: Geschätzte relative Mortalitätsraten (RR) und 95%-Unsicherheitsintervalle (RR 2,5%, RR 97,5%) für das Negativ-Binomial-Regressionsmodell für Mortalitätsdaten von 2015 bis 2019 in Rheinland-Pfalz. GISD: *German Index of Socioeconomic Deprivation*

Koeffizient	RR	RR 2,5%	RR 97,5%
Datum Sinus	1,04	1,03	1,05
Datum Cosinus	0,99	0,97	1,01
GISD	1,63	1,55	1,70
Spline <18°	0,74	0,70	0,78
Spline >18°	0,88	0,82	0,94
Datum	0,99	0,98	0,99
Influenza	1,00	1,00	1,00
Geschlecht M	1,72	1,55	1,91
Alter 0-09	0,92	0,80	1,04
Alter 10-19	0,27	0,22	0,33
Alter 20-29	0,46	0,40	0,54
Alter 40-49	2,34	2,13	2,58
Alter 50-59	6,73	6,17	7,35
Alter 60-69	16,84	15,48	18,35
Alter 70-79	44,79	41,20	48,80
Alter 80-89	151,70	139,69	165,27
Alter 90+	478,37	440,38	521,28
Geschlecht M: Alter 0-09	0,67	0,56	0,79
Geschlecht M: Alter 10-19	0,90	0,70	1,15

Koeffizient	RR	RR 2,5%	RR 97,5%
Geschlecht M: Alter 20-29	1,22	1,02	1,47
Geschlecht M: Alter 40-49	0,99	0,88	1,12
Geschlecht M: Alter 50-59	0,97	0,87	1,08
Geschlecht M: Alter 60-69	1,04	0,93	1,15
Geschlecht M: Alter 70-79	0,97	0,87	1,07
Geschlecht M: Alter 80-89	0,78	0,70	0,86
Geschlecht M: Alter 90+	0,65	0,59	0,72

Tabelle Z3: Gesamte COVID-19 zugeschriebene Sterbefälle und Übersterblichkeit mit 95%-Vorhersageintervallen von 01/2020 bis 07/2021 in deutschen Bundesländern. Die Sterbefälle werden als absolute Anzahl, als Sterbefälle pro 100.000 Einwohner und als Sterbefälle relativ zur Gesamtmortalität (%) angegeben.

		Absolute Todesfälle		Todesfälle/100.000 Einwohner		Todesfälle/Gesamtmortalität [%]	
Bundesland	Bevölkerung	COVID-19	Übersterblichkeit	COVID-19	Übersterblichkeit	COVID-19	Übersterblichkeit
Baden-Württemberg	11.116.092	1.0397	-1.356 [-15.638; 12.147]	93,6	-12 [-141; 109]	5,7	-0,7 [-8,6; 6,6]
Bayern	13.145.594	15.208	9.848 [-6.902; 25.707]	115,7	75 [-53; 196]	6,7	4,4 [-3,1; 11,4]
Berlin	3.674.319	3.501	-1.970 [-6.998; 2.814]	95,4	-53 [-190; 77]	5,9	-3,3 [-11,8; 4,7]
Brandenburg	2.528.639	3.791	1.752 [-2.877; 6.132]	149,8	69 [-114; 242]	6,7	3,1 [-5,1; 10,9]
Bremen	680.411	499	176 [-1.191; 1.473]	73,4	26 [-175; 216]	3,9	1,4 [-9,3; 11,5]

		Absolute Todesfälle		Todesfälle/100.000 Einwohner		Todesfälle/Gesamtmortalität [%]	
Bundesland	Bevölkerung	COVID-19	Übersterblichkeit	COVID-19	Übersterblichkeit	COVID-19	Übersterblichkeit
Hamburg	1.853.220	1.628	2.573<br>[124; 4.885]	87,8	139<br>[7; 264]	5,6	8,9<br>[0,4; 16,8]
Hessen	6.296.061	7.356	7.265<br>[-1.022; 15.109]	116,8	115<br>[-16; 240]	6,6	6,5<br>[-0,9; 13,5]
Mecklenburg-Vorpommern	1.609.047	1.187	-434<br>[-3.650; 2.606]	73,8	-27<br>[-227; 162]	3,3	-1,2<br>[-10,2; 7,3]
Niedersachsen	8.002.109	5.803	4.758<br>[-6.966; 15.880]	72,5	59<br>[-87; 198]	3,8	3,1<br>[-4,5; 10,3]
Nordrhein-Westfalen	17.933.955	17.283	10.524<br>[-14.868; 34.476]	96,4	59<br>[-83; 192]	5,1	3,1<br>[-4,4; 10,1]

		Absolute Todesfälle		Todesfälle/100.000 Einwohner		Todesfälle/Gesamtmortalität [%]	
Bundesland	Bevölkerung	COVID-19	Übersterblichkeit	COVID-19	Übersterblichkeit	COVID-19	Übersterblichkeit
Rheinland-Pfalz	4.097.399	3.714	1.329<br>[-4.931; 7.226]	90,6	32<br>[-120; 176]	4,8	1,7<br>[-6,3; 9,3]
Saarland	984.449	1.003	1.573<br>[-400; 3.443]	101,9	160<br>[-41; 350]	4,6	7,2<br>[-1,8; 15,8]
Sachsen	4.063.732	10.103	6.076<br>[-1.619; 13.333]	248,9	150<br>[-39; 329]	10,2	6,1<br>[-1,6; 13,5]
Sachsen-Anhalt	2.182.831	3.471	3.595<br>[-882; 7.859]	159,3	165<br>[-40; 360]	6,2	6,5<br>[-1,6; 14,1]
Schleswig-Holstein	2.908.823	1.626	-368<br>[-5.124; 4.144]	55,9	-13<br>[-176; 142]	2,9	-0,7<br>[-9,1; 7,3]

		Absolute Todesfälle		Todesfälle/100.000 Einwohner		Todesfälle/Gesamtmortalität [%]	
Bundesland	Bevölkerung	COVID-19	Übersterblichkeit	COVID-19	Übersterblichkeit	COVID-19	Übersterblichkeit
Thüringen	2.123.017	4.373	3.290 [-749; 7.120]	206,3	155 [-35; 336]	8,7	6,6 [-1,5; 14,2]

Tabelle Z4: Gesamte COVID-19 zugeschriebene Sterbefälle und Übersterblichkeit mit 95%-Vorhersageintervallen von 01/2020 bis 07/2021 in den Kreisen von Rheinland-Pfalz. Die Sterbefälle werden als absolute Anzahl, als Sterbefälle pro 100.000 Einwohner und als Sterbefälle relativ zur Gesamtmortalität (%) angegeben.

		Absolute Todesfälle		Todesfälle/100.000 Einwohner		Todesfälle/Gesamtmortalität [%]	
Kreis	Bevölkerung	COVID-19	Übersterblichkeit	COVID-19	Übersterblichkeit	COVID-19	Übersterblichkeit
Ahrweiler	131.465	54	19	41,1	14	1,9	0,7
			[-480; 487]		[-365; 370]		[-17,3; 17,6]
Altenkirchen	128.646	101	56	78,5	43	3,9	2,1
(Westerwald)			[-427; 500]		[-332; 389]		[-16,4; 19,2]
Alzey-Worms	130.402	124	-25	94,9	-19	5,8	-1,2
			[-463; 382]		[-355; 293]		[-21,5; 17,7]
Bad Dürkheim	134.259	150	121	111,7	90	5,4	4,3
			[-369; 576]		[-275; 429]		[-13,2; 20,6]
Bad	159.165	142	88	89,2	55	4,3	2,7
Kreuznach			[-453; 600]		[-285; 377]		[-13,7; 18,2]

Kreis	Bevölkerung	Absolute Todesfälle COVID-19	Absolute Todesfälle Übersterblichkeit	Todesfälle/100.000 Einwohner COVID-19	Todesfälle/100.000 Einwohner Übersterblichkeit	Todesfälle/Gesamtmortalität [%] COVID-19	Todesfälle/Gesamtmortalität [%] Übersterblichkeit
Bernkastel-Wittlich	112.550	62	-54 [-508; 363]	55,1	-48 [-451; 322]	2,8	-2,4 [-22,8; 16,3]
Birkenfeld	81.390	92	98 [-294; 466]	113,0	120 [-361; 573]	4,9	5,2 [-15,6; 24,8]
Cochem-Zell	61.307	61	10 [-322; 314]	99,5	17 [-525; 512]	4,6	0,8 [-24,5; 23,9]
Donnersberg-kreis	75.353	70	28 [-324; 353]	92,8	37 [-430; 468]	4,7	1,9 [-22,0; 23,9]
Eifelkreis Bitburg-Prüm	98.994	31	-75 [-476; 301]	31,3	-76 [-481; 304]	1,7	-4,2 [-26,4; 16,7]

		Absolute Todesfälle		Todesfälle/100.000 Einwohner		Todesfälle/Gesamtmortalität [%]	
Kreis	Bevölkerung	COVID-19	Übersterblichkeit	COVID-19	Übersterblichkeit	COVID-19	Übersterblichkeit
Frankenthal (Pfalz)	48.650	53	-47 [-341; 217]	108,9	-97 [-701; 446]	5,5	-4,9 [-35,7; 22,7]
Germersheim	129.502	126	134 [-295; 534]	97,3	103 [-228; 412]	5,6	6,0 [-13,2; 23,8]
Kaisers-lautern	105.605	83	-45 [-460; 344]	78,6	-43 [-436; 326]	4,2	-2,3 [-23,4; 17,5]
Kaiserslau-tern (Stadt)	99.489	115	61 [-340; 434]	115,6	61 [-342; 436]	6,1	3,2 [-17,9; 22,8]
Koblenz	112.146	154	116 [-321; 517]	137,4	104 [-286; 461]	6,9	5,2 [-14,3; 23,1]

		Absolute Todesfälle		Todesfälle/100.000 Einwohner		Todesfälle/Gesamtmortalität [%]	
Kreis	Bevölkerung	COVID-19	Übersterblichkeit	COVID-19	Übersterblichkeit	COVID-19	Übersterblichkeit
Kusel	69.769	69	85 [-276; 413]	98,8	122 [-396; 592]	4,3	5,3 [-17,3; 26,0]
Landau in der Pfalz	46.921	35	2 [-255; 236]	74,6	4 [-544; 503]	4,4	0,3 [-32,0; 29,6]
Ludwigshafen am Rhein	173.527	336	-64 [-592; 436]	193,3	-37 [-341; 251]	11,2	-2,1 [-19,7; 14,5]
Mainz	214.585	210	2 [-519; 495]	97,9	1 [-242; 231]	7,0	0,1 [-17,3; 16,5]
Mainz-Bingen	212.233	219	73 [-489; 604]	103,2	34 [-230; 285]	6,3	2,1 [-14,0; 17,3]

		Absolute Todesfälle		Todesfälle/100.000 Einwohner		Todesfälle/Gesamtmortalität [%]	
Kreis	Bevölkerung	COVID-19	Übersterblichkeit	COVID-19	Übersterblichkeit	COVID-19	Übersterblichkeit
Mayen-Koblenz	214.514	178	91 [-532; 677]	82,9	42 [-248; 316]	4,3	2,2 [-12,9; 16,4]
Neustadt adW	53.715	38	-22 [-333; 263]	70,7	-41 [-620; 490]	3,4	-2,0 [-30,0; 23,7]
Neuwied	183.577	194	88 [-493; 633]	105,7	48 [-269; 345]	5,3	2,4 [-13,4; 17,2]
Pirmasens	40.319	54	105 [-189; 371]	133,9	261 [-469; 920]	4,8	9,3 [-16,8; 33,0]
Rhein-Hunsrück-Kreis	103.330	103	122 [-300; 509]	99,6	118 [-290; 493]	4,8	5,7 [-14,1; 23,9]

		Absolute Todesfälle		Todesfälle/100.000 Einwohner		Todesfälle/Gesamtmortalität [%]	
Kreis	Bevölkerung	COVID-19	Übersterblichkeit	COVID-19	Übersterblichkeit	COVID-19	Übersterblichkeit
Rhein-Lahn-Kreis	122.235	98	104 [-367; 541]	80,1	85 [-300; 442]	3,8	4,0 [-14,3; 21,1]
Rhein-Pfalz-Kreis	156.022	230	150 [-361; 631]	147,3	96 [-232; 404]	7,6	5,0 [-11,9; 20,9]
Speyer	50.574	85	113 [-168; 366]	167,8	223 [-333; 723]	8,2	10,8 [-16,1; 35,1]
Südliche Weinstraße	110.807	118	-38 [-470; 358]	106,4	-34 [-424; 323]	5,7	-1,8 [-22,8; 17,4]
Südwestpfalz	94.437	85	-88 [-523; 310]	90,0	-93 [-554; 328]	4,2	-4,4 [-26,1; 15,4]

		Absolute Todesfälle		Todesfälle/100.000 Einwohner		Todesfälle/Gesamtmortalität [%]	
Kreis	Bevölkerung	COVID-19	Übersterblichkeit	COVID-19	Übersterblichkeit	COVID-19	Übersterblichkeit
Trier	107.523	30	-86	27,9	-80	1,8	-5,0
			[-479; 278]		[-446; 258]		[-28,0; 16,3]
Trier-Saarburg	150.634	95	-162	63,0	-107	3,6	-6,1
			[-662; 311]		[-439; 207]		[-25,1; 11,8]
Vulkaneifel	60.289	61	22	101,3	36	4,6	1,7
			[-313; 325]		[-519; 539]		[-23,7; 24,6]
Westerwald-kreis	202.655	156	-57	76,9	-28	4,2	-1,5
			[-656; 504]		[-324; 249]		[-17,6; 13,6]
Worms	85.819	94	-56	109,6	-65	6,2	-3,7
			[-425; 282]		[-495; 329]		[-28,1; 18,6]

		Absolute Todesfälle		Todesfälle/100.000 Einwohner		Todesfälle/Gesamtmortalität [%]	
Kreis	Bevölkerung	COVID-19	Übersterblichkeit	COVID-19	Übersterblichkeit	COVID-19	Übersterblichkeit
Zweibrücken	33.699	9	-37	26,7	-110	1,3	-5,5
			[-282; 182]		[-837; 540]		[-41,8; 27,0]

5 Literatur

1. German Federal Statistical Office. Sterbefälle (2021) Fallzahlen nach Tagen, Wochen, Monaten, Altersgruppen, Geschlecht und Bundesländern für Deutschland 2016 – 2021. https://www.destatis.de/DE/Themen/Gesellschaft-Umwelt/Bevoelkerung/Sterbefaelle-Lebenserwartung/sterbefallzahlen.html
Zugegriffen: 3. September 2021.

2. Kroll LE, Schumann M, Hoebel J, Lampert T (2017) Regionale Unterschiede in der Gesundheit - Entwicklung eines sozioökonomischen Deprivationsindex für Deutschland. Journal of Health Monitoring 2:103–20.

3. Robert Koch Institut FG 28 (2021) Soziale Determinanten der Gesundheit. German Index of Socioeconomic Deprivation
https://github.com/GISD-FG28/GISD
Zugegriffen: 24. August 2021.

4. Robert Koch Instititute (2021) SurvStat@RKI 2.0 Influenza Data
https://survstat.rki.de/
Zugegriffen: 3. September 2021.

5. Robert Koch Institute (2021) COVID-19 Todesfälle nach Sterbedatum
https://www.rki.de/DE/Content/InfAZ/N/Neuartiges_Coronavirus/Projekte_RKI/COVID-19_Todesfaelle.html
Zugegriffen: 3. September 2021.

6. Robert Koch Institute (2021) RKI COVID19 Dashboard
https://corona.rki.de/
Zugegriffen: 3. September 2021.

7. Robert Koch Institute (2021) RKI COVID19 Dashboard Data
https://npgeo-corona-npgeo-de.hub.arcgis.com/datasets/dd4580c810204019a7b8eb3e0b329dd6_0/data
Zugegriffen: 3. September 2021.

8. Bürkner PC (2017) brms: An R package for Bayesian multilevel models using Stan. Journal of Statistical Software 80.

9. Carpenter B, Gelman A, Hoffman MD, Lee D, Goodrich B, Betancourt M, et al (2017) Stan: A probabilistic programming language. Journal of Statistical Software 76.
